# Sparse balance: Excitatory-inhibitory networks with small bias currents and broadly distributed synaptic weights

**DOI:** 10.1371/journal.pcbi.1008836

**Published:** 2022-02-09

**Authors:** Ramin Khajeh, Francesco Fumarola, LF Abbott

**Affiliations:** 1 Mortimer B. Zuckerman Mind Brain Behavior Institute, Department of Neuroscience, Columbia University, New York City, New York, United States of America; 2 Laboratory for Neural Computation and Adaptation, RIKEN Center for Brain Science, Saitama, Japan; Dartmouth College, UNITED STATES

## Abstract

Cortical circuits generate excitatory currents that must be cancelled by strong inhibition to assure stability. The resulting excitatory-inhibitory (E-I) balance can generate spontaneous irregular activity but, in standard balanced E-I models, this requires that an extremely strong feedforward bias current be included along with the recurrent excitation and inhibition. The absence of experimental evidence for such large bias currents inspired us to examine an alternative regime that exhibits asynchronous activity without requiring unrealistically large feedforward input. In these networks, irregular spontaneous activity is supported by a continually changing sparse set of neurons. To support this activity, synaptic strengths must be drawn from high-variance distributions. Unlike standard balanced networks, these sparse balance networks exhibit robust nonlinear responses to uniform inputs and non-Gaussian input statistics. Interestingly, the speed, not the size, of synaptic fluctuations dictates the degree of sparsity in the model. In addition to simulations, we provide a mean-field analysis to illustrate the properties of these networks.

## Author summary

A class of models in computational neuroscience that have been successful at describing a variety of effects in the neocortex involve a tight balance between excitatory, inhibitory and unrealistically large external input, without which the model cannot produce robust patterns of activity. In this work, we explore what happens when these inputs are smaller in size, and we provide an alternative solution for recovering robust network activity. This solution relies on broadly distributed synaptic strengths and, interestingly, gives rise to sparse subsets of neurons firing at any given time. Unlike the conventional models, the networks exhibit nonlinear responses to uniform external input.

## Introduction

A typical cortical pyramidal cell receives thousands of excitatory inputs [1] that, without the influence of inhibition, would drive extremely high firing rates. It has been suggested that the inhibition that moderates these rates sets up a balanced condition that causes neurons to operate in a regime where fluctuations, not the mean, of their inputs drive spiking, resulting in irregular sequences of action potentials [2–5]. A number of theoretical models have been developed to address E-I balance and the irregular firing of cortical neurons (see [6] for a review). In the standard balanced models [7, 8], the input to each neuron has three strong components—recurrent excitation, recurrent inhibition and feedforward excitation. These balance automatically as part of the network dynamics, leaving residual fluctuations that drive neuronal firing at reasonable rates. However, there is no evidence for the strong input components predicted by these models [9], and some evidence against them [10–14]. For this reason, we examine the consequences of removing strong feedforward input in balanced models.

In balanced models, synaptic strengths are drawn independently from two probability distributions, one for excitation and another for inhibition. For standard recurrent models to generate spontaneous irregular (chaotic) activity, the synaptic weight distributions must have a variance of order 1/*K*, where *K* is the in-degree, i.e., the number of inputs per neuron from other cells [8, 15–17]. The excitatory (inhibitory) distributions are only non-zero for non-negative (non-positive) values, and typically their mean is of the same order as the square-root of their variance, with both being of order $1/\sqrt{K}$. Summing this mean over the *K* inputs to each neuron gives a total input, which is typically inhibitory, of order $\sqrt{K}$. This large mean input must be cancelled and, in conventional models, this is done by adding constant feedforward excitation that is also of order $\sqrt{K}$. This is the large feedforward input that we aim to avoid.

A first question to ask is what happens if the order $\sqrt{K}$ input is simply left out of the standard models and replaced by an input of order 1. This results in a constraint on the firing rates; specifically, the average firing rate in the network must be of order $1/\sqrt{K}$. This implies that, although there can be irregular spontaneous activity without strong feedforward input, it involves neurons firing at very low rates. One way around this problem is to note that a small average firing rate is not incompatible with having individual neurons with significant firing rates if the activity is sparse. In other words, the average rate can be of order $1/\sqrt{K}$ if, as in the standard models, activity is dense and individual rates are of order $1/\sqrt{K}$, or if individual rates are of order 1 and the fraction of active neurons is of order $1/\sqrt{K}$. Here, we explore this latter possibility.

We mentioned above that standard balanced models require synaptic distributions with variance of order 1/*K* to generate irregular spontaneous activity. More precisely, the requirement is that, for each neuron, the sum of the variances of the strengths of its active inputs must be of order 1. In the standard model, this is satisfied because the product of *K*, the order of magnitude of the number of active inputs, and 1/*K*, the variance per synapse, is 1. In the sparse models proposed in the previous paragraph, the number of active inputs is only of order $\sqrt{K}$, so the total variance computed in this way would be $\sqrt{K}/K=1/\sqrt{K}$, which is not sufficient to generate robust irregular activity. To solve this problem, we consider distributions of synaptic strength with means of order $1/\sqrt{K}$, as in the standard model, but with variances of order $1/\sqrt{K}$, not order 1/*K*. In this case, the total variance is of order $\sqrt{K}/\sqrt{K}=1$, and irregular activity is restored.

Another feature of standard balanced models that seems at odds with the data is that they have attenuated linear responses to input that is uniform across neurons [9, 18]. This linearity is not present in the networks we study. In summary, the combination of small feedforward inputs and broadly distributed synaptic strengths gives rise to a novel E-I regime that exhibits asynchronous irregular sparse firing. In the following, we illustrate prominent features of this regime, such as nonlinear responses to feedforward input and non-Gaussian current distributions, and we highlight the mechanisms that maintain sparsity and distributed firing across network neurons.

Before proceeding, we note that the term ‘balance’ in ‘sparse balance’ refers to a mechanism that constrains input currents to be order 1 despite large numbers of afferents to each neuron. In our model, this constraint is satisfied by sparsity rather than excitatory-inhibitory current cancellation. Thus, models in the ‘sparse balance’ regime are not really balanced, but we retain the term because these models are constrained by that same condition that imposes excitatory-inhibitory balance in the conventional models.

## Results

### The model

A common simplification for analyzing E-I networks is to consider a single population of inhibitory units driven by excitatory input from an external source [16, 17]. After analyzing such purely inhibitory networks, we will show that our results also apply to networks with both excitatory and inhibitory units. We consider standard ‘rate’ models. The inhibitory networks we study have currents *x*_*i*_ for *i* = 1, 2, …, *N* and firing rates *ϕ*(*x*_*i*_) that obey

$$\begin{array}{c} \tau_{x}\frac{dx_{i}}{dt}=-x_{i}-\sum_{j=1}^{N}J_{ij}\phi \left(x_{j}\right)+I_{0}\;, \end{array}$$ (1)

where *ϕ* is a nonlinear function and *J*_*ij*_ ≥ 0. We call the variable *x* a current because it represents the total current generated by the recurrent synaptic and feedforward inputs in the second and third terms on the right side of the above equation, and because it determines the firing rate through the ‘F-I’ function *ϕ*(*x*). We also use the terms ‘response’ or ‘firing rate’ for *ϕ*(*x*) and ‘activity’ for non-zero rates. In our plots, we measure time in units of *τ*_*x*_, making it a dimensionless variable. Connectivity can be all to all (*K* = *N*) or we can restrict the connectivity so that only *K* < *N* of the elements in each row of *J* are non-zero. *I*_0_ is a positive bias input that is identical for all units; it is the feedforward input discussed in the Introduction. Standard balanced models assume the unrealistically large scaling $I_{0}∼\sqrt{K}$; we consider, instead, models with *I*_0_ of order 1.

The non-zero elements of *J* are drawn independently from a distribution with mean $J_{0}/\sqrt{K}$, with *J*_0_ an order 1 parameter. We express the variance of this distribution as *g*^2^/*K*^*ν*^, where *g* is another parameter of order 1, and *ν* allows us to vary the scaling with *K*. The standard scaling is *ν* = 1, which we call low variance. As we will show, the novel sparse balance regime we explore comes about from setting *ν* = 1/2, which we call high variance.

To simplify analysis, synaptic weights in ‘excitatory-inhibitory’ models are sometimes drawn from non-zero mean Gaussians, even though the resulting synapses are not strictly sign-constrained. This is a bigger problem in the large variance cases we study because a considerable portion of the Gaussian distribution generates synapses of the wrong sign. For this reasons, although we discuss an example network with Gaussian weights, we primarily use distributions with exclusively positive support, such as lognormal and gamma, focusing particularly on gamma-distributed synapses for reasons given below. This specific choice is not essential; network behavior remains qualitatively the same across a variety of weight distributions, including binary (S1 Fig) and even Gaussian (S2 Fig).

**Fig 1:**
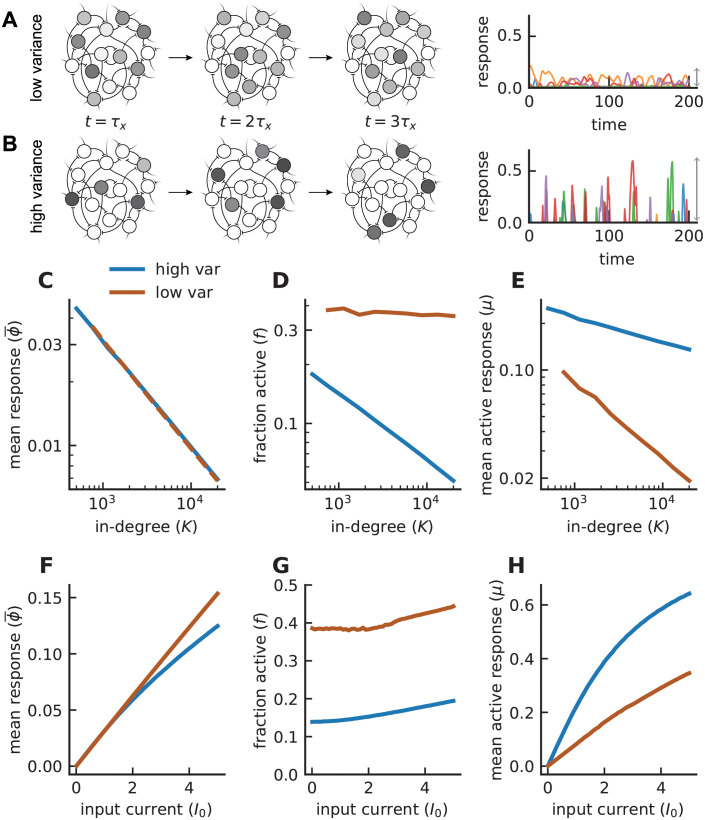
Comparison of low- and high-variance networks. **A)** Cartoon of network dynamics in time. Time is measured in units of *τ*_*x*_. Light (dark) gray corresponds to low (high) firing rates. With low synaptic variance, fluctuations in firing rates are small, and a relatively fixed and dense subset of units contribute to firing. Right: firing rate traces of five example units (each with a distinct color). Gray arrow indicates the extent of fluctuations in the network. **B)** Same as **A** except for the high-variance model. The network exhibits a small and shifting ensemble of cells that respond robustly at any given time. The magnitude of fluctuations is increased substantially (right). **C)** Mean response in both networks follows a $1/\sqrt{K}$ scaling (fits to the data yield $\bar{\phi }∼1/K^{0.503}$ for high variance and ∼ 1/*K*^0.513^ for low variance; *J*_0_ is adjusted so that $\bar{\phi }$ values in the two networks overlaps). **D)** Fraction of active units (inverse sparsity). High-variance model exhibits a rapid sparsening in *K* while, in the low-variance network, this fraction remains roughly constant. **E)** Mean response of the active subset. The trend in **D** is flipped: the low-variance network exhibits a rapidly vanishing *μ*, which is not the case in the high-variance model. Input current *I*_0_ is set to one. **F)** Network’s response to the external input current *I*_0_ with *K* = 1000. **G)** Despite similar $\bar{\phi }$ values, the high-variance network is more sparsely active by more than twofold. **H)** Active neurons respond more robustly in the high-variance network than in its low-variance counterpart. (Model parameters: *J*_0_ = 2 for high variance and 1.05 for low variance, *g* = 2, *J*_*ij*_ ∼ gamma, *N* = *K*, *ϕ* = [tanh]_+_).

We begin (Fig 1) by setting the response nonlinearity *ϕ* to a rectified hyperbolic tangent,

$$\begin{array}{c} \phi \left(x\right)=\{\begin{array}{cc} \text{tanh}(x)\; & x>0\\ 0\; & x\leq 0 \end{array} \end{array}$$ (2)

or

$$\begin{array}{c} \phi \left(x\right)=\{\begin{array}{cc} x^{\lambda }\; & x>0\\ 0\; & x\leq 0 \end{array} \end{array}$$ (3)

focusing, in particular, on the case *λ* = 0 (Heaviside function), but we also consider λ = 1 (rectified linear), and λ = 2 (rectified quadratic).

Throughout, [⋅] denotes averages over units, 〈⋅〉 denotes averages over time, and an overline represents averages across both units and time. For fixed *K*, the results we present are independent of network size *N*, provided that the networks are large enough. For this reason and because we are interested in large *K*, we restrict our studies to the case *K* = *N*, but the results reported extend to partially-connected networks as well (*K* < *N*; S3 Fig).

### Simulation results

With the usual $I_{0}∼\sqrt{K}$ bias reduced to an input of order 1, the network behaves very differently in the low- (*ν* = 1) and high- (*ν* = 1/2) variance cases (Fig 1). For low variance, many units are active, but their responses are small (Fig 1A). In contrast, for high variance, activity in the network is sparse but individual units exhibit robust responses (Fig 1B). Scaling of the firing rate as a function of connectivity *K* can be quantified by computing

$$\begin{array}{c} \bar{\phi }=\left[\left\langle \phi \right\rangle \right]=\frac{1}{N}\sum_{j=1}^{N}\left\langle \phi_{j}\right\rangle \;. \end{array}$$ (4)

We can break down this average by writing it as the product of *f*, the fraction of units that are active (*ϕ* > 0), and *μ*, the average firing rate of the active units, $\bar{\phi }=f\mu$. In both the low- and high-variance cases, the average firing rate $\bar{\phi }$ scales as $1/\sqrt{K}$ (Fig 1C) but, for low variance, *f* is fairly independent of *K* (Fig 1D) and *μ* scales as $1/\sqrt{K}$ (Fig 1E). The scaling is different for high variance where *f* scales closer to $1/\sqrt{K}$ (Fig 1D) and *μ* is relatively independent of *K* (Fig 1E). Thus, the high-variance case, which we call sparse balance, results in networks in which activity is sparse but individual units have appreciable responses.

A well-known distinctive feature of standard balanced networks ($I_{0}∼\sqrt{K}$ and *J*-variance ∼ 1/*K*) is that the average firing rate $\bar{\phi }$ is a linear function of the bias input *I*_0_ despite the presence of a nonlinear response function in the model. This feature extends to the low bias model (*I*_0_ ∼ 1) in the case of low variance but, for high *J*-variance ($∼1/\sqrt{K}$), the average response $\bar{\phi }$ has a nonlinear dependence on *I*_0_ (Fig 1F). In both the low- and high-variance cases, *f* is insensitive to *I*_0_ (Fig 1G), meaning that the mean firing rate of the active units *μ* is also linear for low variance and nonlinear for high variance (Fig 1H). Thus, the restriction to linear population responses for uniform input does not apply to the sparse balance networks.

**Fig 2:**
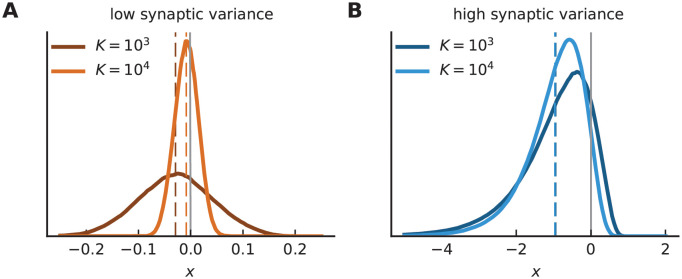
Sparse balance yields non-Gaussian dynamics and a subthreshold mean. Distribution of currents *x* (over time and units) for gamma-distributed synapses. Dashed lines denote the mean of each distribution, i.e., $\bar{x}$. Area above threshold (set to zero; solid line) corresponds to the fraction of active units *f*. **A)** With low synaptic variance (*ν* = 1), the distribution of *x* is a Gaussian centered around a mean that tends to zero for larger *K*. **B)** Same as in A except for high synaptic variance (*ν* = 1/2). Note the larger range of the horizontal axis compared to B. The distribution is no longer Gaussian. $\bar{x}$ is relatively insensitive to *K* and lies below threshold. (Model parameters are *g* = *J*_0_ = 2, *I*_0_ = 1, *J*_*ij*_ ∼ gamma, *N* = *K*, *ϕ* = [tanh]_+_).

We also examined the distribution of *x* values in these networks (Fig 2). In the low-variance case, these distributions are Gaussian, and both their mean and variance decrease in magnitude with *K* (Fig 2A). The result of these two effects is that the fraction of the *x* distribution above threshold (area under the *x* distribution above *x* = 0) remains fairly constant as a function of *K*, corresponding to the roughly constant fraction of active units (Fig 1D). For high variance (Fig 2B), the distribution is non-Gaussian, and the fraction above threshold drops with *K*, again corresponding to the dependence of the average firing-rate response on *K* (Fig 1D and 1E). The mean of the *x* distribution for the sparse balance network is insensitive to *K* and lies below threshold. The mean of the distribution for low variance is also negative, but it moves toward zero as *K* increases.

**Fig 3:**
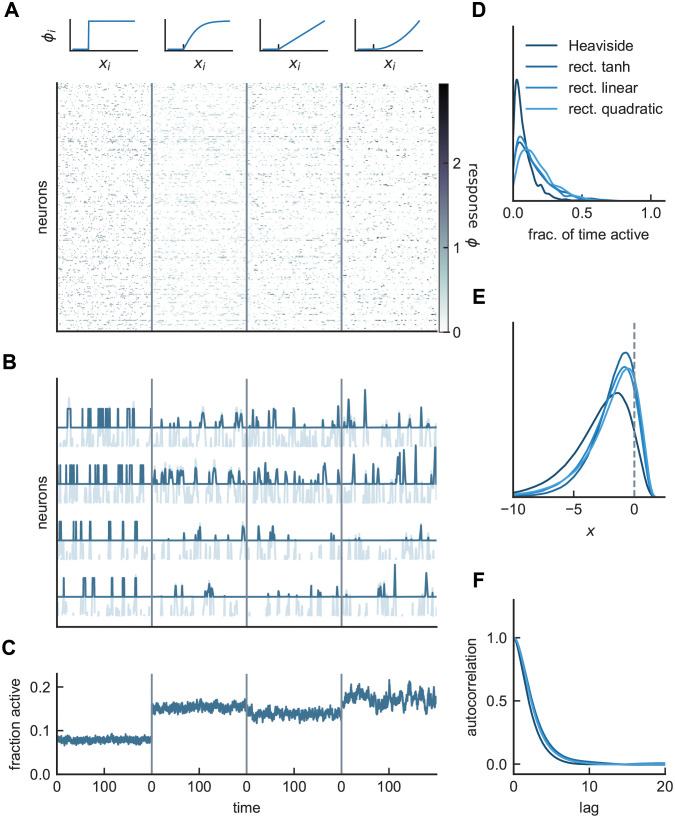
Asynchronous irregular activity in the sparse balance model. **A)** Responses of network neurons in time for four different nonlinear response functions: Heaviside step function, rectified tanh, rectified linear, and rectified quadratic. **B)** Rates *ϕ*(*x*) (dark) superimposed on the currents *x* (light) for four example units. Cells respond robustly and infrequently across choices of the response functions. The synchrony index, as defined in [19], is approximately 10^−4^ for each of the networks shown. **C)** Fractions of active neurons, or the inverse sparsity. **D)** Normalized distributions for the fraction of ON-time, defined as the fraction of (simulation) time a unit spends above threshold. For better visualization, histograms are smoothened using kernel density estimation. **E)** Normalized distributions of *x*, showing non-Gaussian dynamics. **F)** Population-averaged autocorrelation functions of *x*. At this fixed value of in-degree (*K* = 1000), all response functions produce qualitatively similar results. (Model parameters: *g* = *J*_0_ = *I*_0_ = 2, *J*_*ij*_ ∼ gamma, *N* = *K*).

The results for networks with small input biases and large synaptic-weight variances, shown in Fig 1 for a rectified hyperbolic tangent nonlinearity, extend to other nonlinear response functions as well (Fig 3A and 3B). The response in these networks is distributed across almost all of the units, but at any given time only a sparse distinct subset of units is active (Fig 3C). This active population constantly changes, and firing rates appear chaotic. The fraction of time that units are active is skewed toward small values (Fig 3D), indicating that the majority of units respond infrequently. For all choices of *ϕ*, the distribution of *x* is non-Gaussian with only a small fraction of units above threshold (Fig 3E), consistent with the sparsity of the firing. Finally, the dynamics in these networks can be characterized by the population-averaged autocorrelation function of the currents (Fig 3F), which we consider in more detail in a later section.

The intuitive picture given in the introduction and the simple scaling results for the high-variance model (*ν* = 1/2) are based on treating the firing rates as binary, which is only strictly true if the response is given by a Heaviside function. For a Heaviside, the firing rate of an active unit is always one, so *μ* = 1 and the average response is equal to the sparsity, $\bar{\phi }=f$. For other nonlinearities, these simple identities are violated to some degree. In the case of the rectified hyperbolic tangent function in Fig 1, the $1/\sqrt{K}$ scaling of $\bar{\phi }$ is dominated by the scaling of *f*, and not *μ*, but both factors have some *K* dependence. For λ ≥ 2, this is not necessarily the case and *μ* can exhibit a non-negligible scaling with *K* (see S4 Fig). Nevertheless, for *K* ∼ 10^3^ (Fig 3), the responses in the high-variance case are much sparser and more robust than those of its low-variance counterpart, and the low-variance model is prone to entering fixed point states for λ > 1.

The scaling results provided above also extend to a model with Gaussian synapses (S2 Fig). Although synapses in this model are not sign-constrained, this result shows that it is the high variance feature that drives the effects we report. Although, as we discuss below, the sign-constrained models we consider have an interesting feature of non-Gaussian input current statistics, this is not essential to the dynamic regime that characterizes sparse balance. With Gaussian synapses, which are not sign-constrained, the current distribution is also Gaussian, in which case the model is solvable using the standard techniques of dynamic mean-field theory [16, 17]. We present results from such a mean-field analysis in a later section.

In summary, these simulations illustrate an alternative regime for E-I networks in which the activity of individual units remains robust, despite the absence of order $\sqrt{K}$ feedforward bias inputs. Furthermore, in these networks, mean firing rate exhibits a nonlinear dependence on bias input. We now analyze in detail the features illustrated in these simulations.

### Analysis of sparse balance networks

How does high-variance connectivity support sparse but robust activity with low bias input, and what is the nature of this activity? Addressing these questions is simplified by considering a Heaviside response function (Eq (3) with λ = 0; but also see S4 Fig). We consider a general *J*-variance scaling, 1/*K*^*ν*^, so that we can compare results to the low-variance *ν* = 1 case, but we are primarily interested in the high-variance case *ν* = 1/2.

We begin the analysis by defining the recurrent synaptic input as

$$\begin{array}{c} \eta_{i}\left(t\right)=\sum_{j=1}^{N}J_{ij}\;\phi \left(x_{j}\right)\;, \end{array}$$ (5)

so that Eq (1) can be written as

$$\begin{array}{c} \tau_{x}\frac{dx_{i}}{dt}=-x_{i}\left(t\right)-\eta_{i}\left(t\right)+I_{0}\;. \end{array}$$ (6)

Independent of our choice of the synaptic weight distribution, if we average the equation above over both neurons and time and use $\bar{\eta }=J_{0}f\sqrt{K}$, we obtain

$$\begin{array}{c} \bar{x}=-J_{0}f\sqrt{K}+I_{0}\;. \end{array}$$ (7)

Requiring that the total current $\bar{x}$ be of order 1 for large *K* suggests that the firing rates adjust so that $f∼1/\sqrt{K}$. In addition to the mean, we require that the input fluctuations remain finite for large *K*. The total variance of the synaptic input is var(*η*) = *g*^2^ *fK*^1−*ν*^, leading us to conclude that for large *K*, only in the high-variance case will fluctuations remain finite and, for the Heaviside nonlinearity, *ν* must be set to 1/2. The total variance is composed of temporal and ‘quenched’ variances. The first of these variances arises from temporal fluctuations due to the chaotic nature of the dynamics. The quenched variance arises because different units of the network fluctuate around different time-averaged values. Unless specified otherwise, we use ‘variance’ to refer to the total variance, but in a later section we discuss the scaling of these two components of variance separately.

From Eq (7), we can write

$$\begin{array}{c} f=\bar{\phi }=\frac{I_{0}-\bar{x}}{\sqrt{K}\;J_{0}}\;. \end{array}$$ (8)

In the standard balanced model and in the low-variance case considered above, $I_{0}\gg \bar{x}$, so the mean response is linear in *I*_0_. This is no longer true when the synaptic variance is high (*ν* = 1/2) because, in this case, *I*_0_ and $\bar{x}$ are both of order 1. The nonlinear mean response seen in Fig 1F arises because the dependence of $\bar{x}$ on *I*_0_ is nonlinear. In general, the form of this nonlinear response reflects the nonlinearity of *ϕ*, and the sparse balance model can exhibit either sublinear or supralinear mean population responses (S5 Fig). For instance, the sublinearity of the population response observed in Fig 1F reflects the shape of the rectified hyperbolic tangent function and, more generally, the supra/sublinearity of the population response matches the supra/sublinearity of the response function.

For further analysis, we choose a particular choice of connectivity distribution, non-zero weights drawn from a gamma distribution, gamma(*κ*, *θ*), where *κ* and *θ* are the ‘shape’ and ‘scale’ parameters of the gamma distribution in terms of which its mean is *κθ*, and its variance is *κθ*^2^. To achieve a mean $J_{0}/\sqrt{K}$ and variance *g*^2^/*K*^*ν*^ we set

$$\begin{array}{c} \kappa =\frac{J_{0}^{2}}{g^{2}}K^{\nu -1},\;\;\theta =\frac{g^{2}}{J_{0}}K^{1/2-\nu }\;. \end{array}$$ (9)

For a Heaviside nonlinearity, the sum in Eq (5) is only over active units with *ϕ* = 1, and the probability of a unit being active is equal to the sparsity *f*. This means that of the *K* non-zero elements of *J* for each unit, *fK* will be active. As a result, assuming small temporal fluctuations in *f* (Fig 3C), *η* is given by the sum of *fK* random variables drawn independently from the distribution gamma(*κ*, *θ*). The sum of random variables that are gamma-distributed with the same scale parameter is itself gamma-distributed with that scale parameter and a shape parameter equal to the sum of the shape parameters of the variables being summed [20]. Thus,

$$\begin{array}{c} \eta ∼\text{gamma}(\alpha ,\theta )\; \end{array}$$ (10)

with *α* = *fKκ*, which has mean

$$\begin{array}{c} \left[\eta \right]=\alpha \theta =fK\kappa \theta =J_{0}f\sqrt{K} \end{array}$$ (11)

and variance

$$\begin{array}{c} \text{var}\left(\eta \right)=\alpha \theta^{2}=fK\kappa \theta^{2}=g^{2}fK^{1-\nu }\;. \end{array}$$ (12)

Revisiting the result found above, to maintain a finite mean input as *K* grows, Eq (11) implies that we must have $f∼1/\sqrt{K}$, which implies, from Eq (12), that the fluctuations in the synaptic input scale as *K*^1/2−*ν*^. Thus, the only solution with finite fluctuations as *K* grows is the high-variance case, *ν* = 1/2. For *ν* = 1/2 and with $f∼1/\sqrt{K}$ Eqs (11) and (12) show that the distribution of synaptic inputs is independent of *K* (S6 Fig); specifically, both the shape (*α*) and scale (*θ*) parameters of the synaptic input distribution are order 1. This feature, which implies that the distribution of the synaptic input scales as *K*^0^, may appear surprising given that the sparseness of network activity is proportional to $1/\sqrt{K}$. We resolve this paradox in a later section.

A naive application of the central limit theorem would suggest that for sufficiently large *K*, the synaptic input would be normally distributed even though the underlying connectivity is non-Gaussian. But this application rests on the assumption that higher moments of the distribution decrease sufficiently rapidly with increasing *K*, something that is violated in our large-variance case. Independent of *θ*, the larger the shape parameter, the closer a gamma distribution approximates a Gaussian (in particular, the approximation is good for shape parameters ∼20 or larger). The shape parameter for the distribution of *η*, from Eq (10), is $fK\kappa =fJ_{0}^{2}g^{-2}K^{\nu }∼K^{0}$. Thus, unless *J*_0_ is large or *g* is small, even for large *K*, the *η* distribution remains non-Gaussian (Fig 2B and S6 Fig). In the low-variance case, with *ν* = 1 and *f* of order 1, the shape parameter of the *η* distribution is ∼*K*, which explains the Gaussian distribution of the total currents in this model (Fig 2A).

In summary, these analyses show that when feedforward bias input is of order one, large synaptic variance is required to generate robust fluctuations, with a synaptic weight variance of order $1/\sqrt{K}$ producing order-one input fluctuations.

### Sparse activity arises from network dynamics

We noted in the previous section that the distribution of synaptic inputs is independent of *K*, and yet the mean network firing rate $\bar{\phi }$, which is linked to the total current *x* and ultimately to the distribution of synaptic input, varies as $1/\sqrt{K}$. Network currents (*x*) are generated through Eq (6), which involves low-pass filtering of the synaptic input. This suggests that the response sparseness is related not to the distribution of synaptic inputs but rather to their dynamics.

**Fig 4:**
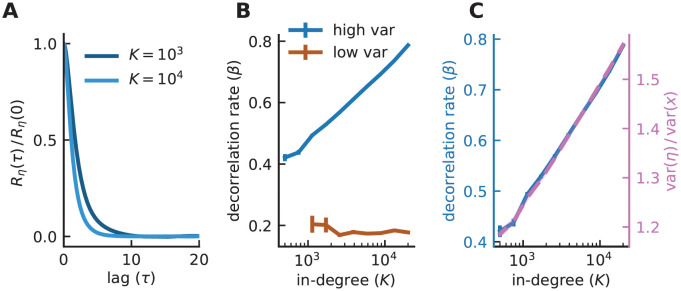
Time-scale of fluctuations adjusts to maintain sparse activity. **A)** Population-averaged autocorrelation function of the synaptic input normalized by its zero-lag value. Note the faster decay of the autocorrelation for increasing *K*. **B)** The decorrelation rate *β* is constant in the low-variance network but increases logarithmically with *K* in the sparse balance model, resembling the (inverted) trends of sparsity (Fig 1D). **C)** *β* (solid) and the ratio var(*x*)/var(*η*) (dashed) in the high-variance model plotted on the same panel, aligned to different y-axes. var(⋅) refers to the total variance; similar result is obtained for the temporal variance. Error bars indicate SEM, averaged over 10 random realizations of the connectivity. (Model parameters: *J*_0_ = 2 for high variance and 1.05 for low variance, *g* = 2, *I*_0_ = 1, *J*_*ij*_ ∼ gamma, *N* = *K*, *ϕ* = [tanh]_+_).

To explore these dynamics, we consider the population-averaged autocorrelation function of *η*,

$$\begin{array}{c} \begin{array}{c} R_{\eta }\left(\tau \right)=[\langle \left(\eta_{i}\left(t\right)-\left\langle \eta_{i}\right\rangle \right)\left(\eta_{i}\left(t+\tau \right)-\left\langle \eta_{i}\right\rangle \right)\rangle ] \end{array} \end{array}$$ (13)

which captures the extent to which *η* at time *t* + *τ* is related to *η* at time *t*. *R*_*η*_ is a decaying function of the lag *τ* (Fig 4A), and its decay defines a correlation time-scale denoted by *τ*_*η*_. One way to define this correlation time-constant is by considering the normalized area underneath the autocorrelation function,

$$\begin{array}{c} \tau_{\eta }=\frac{1}{R_{\eta }\left(0\right)}\int_{0}^{\infty }d\tau R_{\eta }\left(\tau \right) \end{array}$$ (14)

We characterize the dynamics of *η* using the dimensionless constant *β* = *τ*_*x*_/*τ*_*η*_, and find that *β* increases logarithmically with *K*, an increase that does not occur in the low-variance (Fig 4B) or for conventional balanced networks. As *K* increases, the temporal fluctuations in *η* becomes more rapid, although their variance (the temporal variance) remains constant. This makes it increasingly harder for *x* to keep up with the fluctuations, due to the low-pass filtering in Eq (6), leading to a decrease in the temporal variance of *x*. As a result, the fraction of *x* above threshold decreases and the overall activity decreases with *K*. Thus, interestingly, it is the dynamics of the recurrent synaptic inputs, not their size, that leads to sparse activity at large *K*.

To show that the speed of fluctuations in *η*, but not their size, leads to a narrowing of the *x* distribution for increasing *K*, we examine the ratio var(*η*)/var(*x*) (Fig 4C). This quantity, which is the ratio of the total input (*η*) variance to the total output (*x*) variance for the low-pass filter in Eq (6), characterizes the dampening of high-frequency response and thus should be larger for faster input fluctuations even if their size remains constant. Similar to *β* and consistent with the argument above, we observe that var(*η*)/var(*x*) exhibits a logarithmic growth with *K*. Thus, the more rapid temporal fluctuations in *η* lead to a reduction of the temporal variance of *x* and, consequently, to a smaller fraction of activity above threshold, which matches the behavior of the mean response and sparsity.

The quenched variance, which characterizes the width of the distribution of time-averages in the network, is the same for the total current *x* and the synaptic input *η* because Eq 6 only affects the temporal component of the fluctuations. As a result, the behavior of var(*η*)/var(*x*) discussed above is purely due to differences in the time-scale of fluctuations in *η* and *x*. The quenched variance of *x* varies as $∼1/\sqrt{K}$ in the high-variance model (S7 Fig). When $\bar{x}<0$, this supports sparse firing because time-averages of *x* become more narrowly distributed around $\bar{x}$ and are thus forced below threshold with increasing *K*.

As stated earlier, one consequence of the speedup in fluctuation dynamics with *K* is a reduction in the total variance of *x*. In order for the mean response to scale as $1/\sqrt{K}$, this reduction, relative to $\bar{x}$, the mean of *x*, must scale appropriately with *K*. In the Methods, we use a mean-field analysis to show, for the case of a Heaviside response function and Gaussian connectivity, that this scaling is, to leading order, $\text{var}\left(x\right)/{\bar{x}}^{2}∼1/\text{log}K$.

Altogether, these results highlight how the time-scale of synaptic fluctuations dynamically adjust to maintain sparse activity.

### Mean-field analysis

In this section, we present the results of a dynamic mean-field analysis of the sparse balance regime. To make this computation tractable, we consider a network with synapses drawn from a Gaussian distribution. This is useful for the mean-field analysis because the resulting synaptic input and the total current also become Gaussian distributed. As mentioned earlier, this network is not purely inhibitory because, with both synaptic mean and variance of order $1/\sqrt{K}$, a substantial number of synapses will be positive. Nevertheless, this is an instructive example as it highlights that the large scaling of synaptic variance together with order 1 input current are the drivers of the results we report.

**Fig 5:**
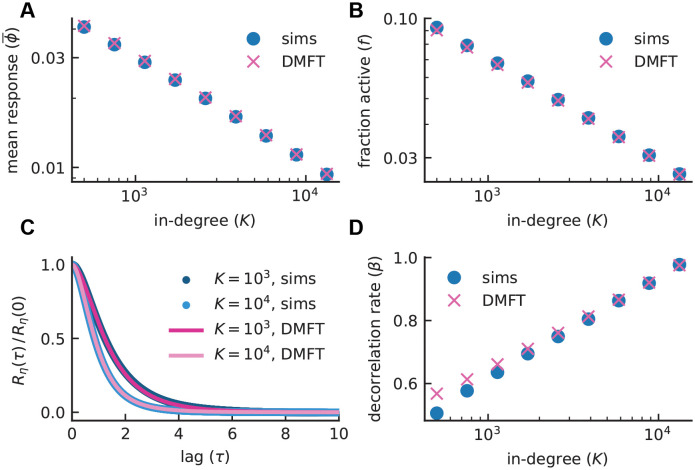
Comparison between network simulations and dynamic mean-field theory (DMFT). Mean response **(A)** and fraction active **(B)** as functions of in-degree *K*. A crucial feature of the sparse balance model is the rapid decay of mean response and sparsity, but not the mean active response (Fig 1E), with increasing *K*. **C)** Autocorrelation of the synaptic input *R*_*η*_(*τ*), normalized by its zero-lag value, exhibits more rapid decorrelation for larger *K*. **D)** *β* grows logarithmically as a function of *K*. Blue data points illustrate averages over 10 random realizations of the connectivity; SEM error bars fall roughly within the size of data points. Crosses indicate self-consistent solutions to Eqs (23)–(25) and show good agreement with the simulation results. (Model parameters: *J*_0_ = 3, *g* = 2, *I*_0_ = 1, *J*_*ij*_ ∼ Gaussian, *ϕ* = [tanh]_+_).

Using standard techniques of dynamic mean-field theory (see Methods; [16, 17, 21]), we calculated mean-field approximations for the mean response $\bar{\phi }$ (Fig 5A), fraction of active neurons *f* (Fig 5B), and the autocorrelation function of the synaptic input *R*_*η*_(*τ*) (Fig 5C), which was then used to compute the decorrelation rate *β* (Fig 5D). The theory is in good agreement with numerical simulations and captures the rapid falloff of the mean response and fraction active as well as the logarithmic increase of *β* with *K*.

### Sparse balance in an E-I network

**Fig 6:**
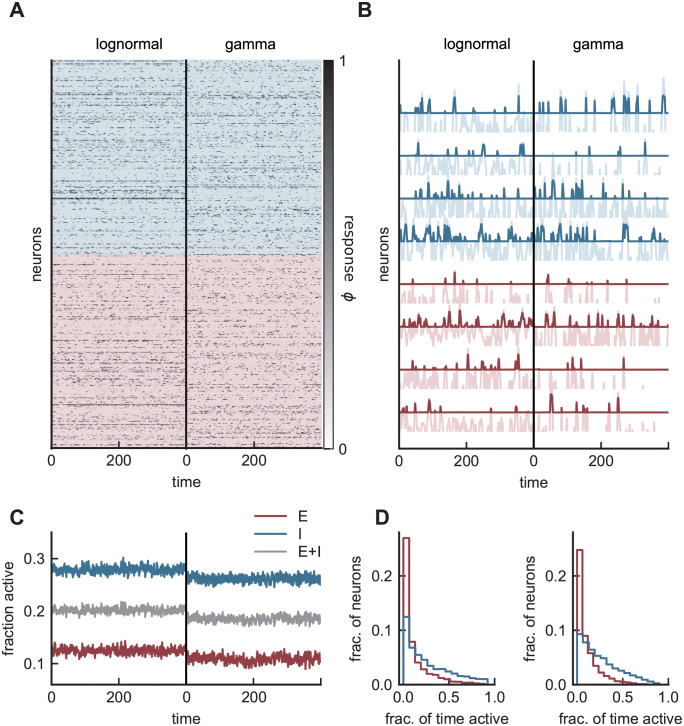
Asynchronous irregular activity in an E-I network with small input current. **A)** Responses of 500 excitatory (red) and 500 inhibitory (blue) units in two networks, one with lognormal (left) and the other with gamma (right) weight distributions. Responses are sparse and distributed across the population. **B)** Rates (dark) superimposed on the currents (light) for four example cells from each population. Response is infrequent as fluctuations occasionally push the current above threshold. **C)** Fraction of active units for individual populations (red and blue) and across the entire network (gray). The inhibitory population is more active than its excitatory counterpart. **D)** Fraction of (simulation) time units spend above threshold for each population and connectivity distribution. This distribution is wide and skewed. Both choices of the connectivity distribution produce qualitatively similar results. (Model parameters: *g* = 1, *J*_*EE*_ = *J*_*IE*_ = 1, *J*_*EI*_ = 2, *J*_*II*_ = 1.2, *I*_*E*_ = 2, *I*_*I*_ = 1, *N*_*E*_ = *N*_*I*_ = 3000, *K* = 600, *ϕ* = [tanh]_+_).

Finally, we illustrate that all of the features we have discussed for a purely inhibitory network are present in mixed excitatory-inhibitory networks for two choices (gamma and lognormal) of the connectivity distribution (Fig 6). These networks exhibit asynchronous irregular activity with chaotic responses of individual units (Fig 6A and 6B) and constant population activity (Fig 6C). Responses are sparse across the population: roughly 10% of excitatory and 20–30% of inhibitory units are active at any given time (Fig 6C). Individual units show sporadic response for both E and I cells in time (Fig 6B) with spatiotemporal variability that is purely internally generated.

Responses are robust and shared across the entire population as opposed to a fixed subset of units. We characterize this feature by considering the distribution of ON-time fraction, i.e., the fraction of time individual cells spend above threshold (Fig 6D). This quantity shows a wide and skewed distribution across both E and I populations. The majority of units spend very little time above the threshold, with only a few (5% of E, 20% of I cells with lognormal; 2% of E, 15% of I cells with gamma) spending more than half the time above threshold, and none responding at all times. We note that gamma and lognormal synaptic distributions produce similar activity patterns across the population.

## Discussion

We have uncovered a novel regime of E-I networks that exhibits asynchronous irregular activity without the need for unrealistically large external input currents. We have done so by taking advantage of widely distributed synapses that generate fluctuations that would otherwise be minuscule in the absence of large feedforward currents. We highlighted a number of properties including sparse activity, non-Gaussian dynamics and a nonlinear population response. Additionally, we showed that the speed, not the size, of input fluctuations is the driver of sparse network activity. Using dynamic mean-field theory, we computed the steady-state distribution and temporal correlations of the recurrent input in a network with normally-distributed synapses. In general, this work further demonstrates the important role of synaptic variance in the dynamics of recurrent networks.

Robust network responses with small input currents are especially interesting in light of the fact that experiments suggest that the feedforward component of the input in cortical circuits is comparable in strength to the total synaptic input (see [9] for a review). For example, [10] and [11] observed that the voltage response with cortex silenced was smaller (about a third) than the response with cortex intact, not larger as would be predicted for the standard balanced state. Additional evidence for small feedforward current stems from experiments in the primary visual cortex of mice in which optogenetic silencing of intracortical excitatory input isolated the feedforward (thalamic) component of the net excitation [13, 14]. The reported thalamic currents (60–150 pA) would, for a typical membrane resistance (∼ 200 MΩ), induce a depolarization of 12–30 mV, comparable to the typical distance to threshold (∼ 10–20 mV) and thus of order 1 [9, 13, 14]. Similar results have been reported in the auditory cortex [22]. Pharmacological silencing of intracortical activity in olfactory cortex (piriform) revealed that odor-evoked feedforward currents (∼ 60 pA) originating from the olfactory bulb also generate depolarizations (∼ 12 mV) comparable to the typical distance to threshold [12].

To provide a more quantitative link to these experimental findings, it is useful to define $\chi =\bar{x}/I_{0}$, referred to as the ‘balanced index’ [9]. The ratio *χ* captures the relative contribution of the feedforward input *I*_0_ to the mean of the total current $\bar{x}$. The aforementioned experiments suggest a *χ* of order 1. In both the standard balanced and the low-variance networks result in $\chi ∼1/\sqrt{K}$. In the sparse balance model, widely distributed synapses together with small input currents yield a *χ* of order 1 in agreement with experimental findings in cortex.

The mean of the total current in the sparse balance model, $\bar{x}$, comprised of the feedforward input and the mean recurrent input, lies well below threshold. One consequence of this, as mentioned above, is that the net input and the feedforward input have comparable contributions to the mean response. This results in a nonlinear population response to uniform input that is absent in the standard balanced regime where the strength of the feedforward input is much larger than the net input. Nonlinear mean responses are known to be necessary for a variety of cortical computations such as response normalization and surround suppression in visual cortex [9, 18, 23–25] and concentration invariance in olfactory cortex [26–28]. In the sparse balance model, the shape of this nonlinear population response depends on the choice of neuronal response function.

Small input currents impose a constraint on the mean response. To ensure this constraint is carried over to the sparsity, but not the mean response of the active neurons, we considered widely distributed synapses through an unconventional scaling of synaptic variance. To ensure sign-constrained synapses, it is natural to use a heavy-tailed synaptic distribution. Models that address the role of heavy-tailed connectivity distributions are timely because it has been shown that the distribution of synaptic efficacies in cortex are compatible with a lognormal fit [1, 29, 30]. Experiments and modeling studies have also suggested that strong synapses in the tail of such distributions, although less frequent, can have a strong influence on postsynaptic firing and network dynamics [31–34].

Our choice of the gamma distribution, as opposed to the lognormal, was motivated by its analytical tractability. This choice was convenient because with Heaviside units the synaptic currents are also distributed according to a gamma distribution. The central limit theorem does not apply to the synaptic input distributions we used, so they remain non-Gaussian even for large in-degree. Furthermore, the scaling of variance we considered results in effectively sparse connectivity in which the majority of synapses are weak and neuronal activity is heavily influenced by a minority of strong synapses. Related to this idea, recent modeling work has demonstrated that networks with power-law synaptic weights exhibit self-sustained activity [34]. In these ‘Cauchy networks’, the variance of the connectivity is infinite for finite *K*, and network behavior is dominated by large tails in the weight distribution. A similar connectivity distribution in spiking models exhibits nonlinear response and high firing rates in response to input currents [35]. In another modeling study, a lognormal distribution of synaptic weights in a network of spiking neurons gave rise to self-sustained asynchronous firing in the absence of any bias input current [32]. Together with these results, our model highlights the degree to which synaptic variance and heterogeneity in connectivity can compensate for the absence of large input currents and sustain rich network dynamics.

The emergence of sparse activity in our model is interesting given that only a small fraction of cortical cells, particularly in the superficial layers, are active in response to many stimulus or spontaneously (see [36] for a review). The fraction of active neurons in our model is predicted to be $∼1/\sqrt{K}$. Pyramidal cells receive ∼7000 excitatory synapses [1], but when we account for the average number of synapses per connection, ∼4 [37, 38] and the number of non-intracortical synapses, the in-degree suggested for our model is *K* ∼ 10^3^. This implies a sparsity of order 3%, a reasonable value.

In our model, the time-scale of the synaptic input, not its distribution, adjusts to maintain the degree of sparsity in the network. As *K* increases, fluctuations of the synaptic input speed up and, because the unit dynamics is a low-pass filter of the synaptic input, the distribution of *x* narrows and the amount of activity above threshold is reduced in a manner consistent with the constraint on the mean rate. Changes in sparsity are made possible by changes in the autocorrelation time-scale, and this phenomenon appears to apply generally. This feature highlights the flexibility of recurrent dynamics in adjusting not only the mean and variance of the distribution of its firing rates, but also their correlation time-scales.

Mean-field theory is an important tool for understanding network behavior. We examined a dynamic mean-field theory in the sparse balance model with Gaussian-distributed synapses. Although this model is not strictly sign-constrained, the scaling of the mean response, sparsity and fluctuation speeds, match those of the model with gamma-distributed synapses, demonstrating that the driver of these effects is high synaptic variance together with small input current. The theory accurately predicted steady-state and temporal correlation of the network dynamics.

One prominent theory that addresses the issue of bias input is the stabilized supralinear network (SSN) [9, 25, 39]. Important features of SSNs include supralinear neuronal response function, small bias current, and weak synaptic coupling. These models have been extensively and successfully used to describe steady-state responses in sensory cortex. Our model differs from the SSN in that it generates chaotic activity, has strong synaptic coupling [40] and has widely distributed synaptic weights. With a supralinear response function (λ > 1), the large synaptic variance endows our model with a higher degree of chaos than standard strongly-coupled networks; without this large degree of heterogenity in the weights, responses are susceptible to resting at fixed points [16]. This degree of chaos may aid in the learning of functional trajectories [41–45].

We examined a model with random connectivity, but it would be interesting to investigate stimulus selectivity in sparse balance networks with structured connections. The large degree of variability in the synapses could route stimulus information along particular paths across network neurons. Structured connectivity is of particular interest given compelling evidence that the recurrent contribution of the synaptic input, not just the feedforward component, exhibits selectivity [12–14]. We believe that the variance of connectivity, in addition to its mean structure, is important to consider for addressing the way feedforward and recurrent components shape selective responses.

## Methods

### Dynamic mean-field theory for the sparse balance model with Gaussian synapses

We consider the dynamic mean field theory for a single population of neurons in the sparse balance state. Synapses in the model are drawn from negative-mean Gaussians, specifically with mean $J_{0}^{2}/\sqrt{K}$ and variance $g^{2}/\sqrt{K}$. Although this model resembles a purely inhibitory network, given the broad distribution of synapses the neurons are not strictly sign-constrained. The analytical match to the theory, for *ϕ* set to rectified tanh, is presented in Fig 5.

The equation describing the full model can be written as

$$\begin{array}{c} \frac{dx_{i}\left(t\right)}{dt}=-x_{i}\left(t\right)-\eta_{i}\left(t\right)+I_{0}\;,\;\;\eta_{i}\left(t\right)=\sum_{j=1}^{N}J_{ij}\;\phi \left(x_{j}\right)\;. \end{array}$$ (15)

The mean-field model involves averages over both temporal fluctuations, denoted by 〈⋅〉, and quenched fluctuations, denoted by [⋅].

We begin by calculating the mean and quenched variance of the synaptic input *η* using standard mean-field approximations. The mean of *η* is [〈*η*〉] = *K*[*J*][〈*ϕ*〉] = $J_{0}\sqrt{K}\left[\langle \phi \rangle \right]$. The quenched variance of *η* is

$$\begin{array}{c} \begin{array}{cc} {}[{\left\langle \eta \right\rangle }^{2}]-{\left[\left\langle \eta \right\rangle \right]}^{2} & =[\sum_{j}\sum_{k}J_{ij}J_{ik}\left\langle \phi_{j}\right\rangle \left\langle \phi_{k}\right\rangle ]-{\left[\sum_{j}J_{ij}\;\left\langle \phi_{j}\right\rangle \right]}^{2}\\ & =K[J^{2}][{\left\langle \phi \right\rangle }^{2}]+(K^{2}-K){\left[J\right]}^{2}{\left[\left\langle \phi \right\rangle \right]}^{2}-K^{2}{\left[J\right]}^{2}{\left[\left\langle \phi \right\rangle \right]}^{2}\;. \end{array} \end{array}$$ (16)

Inserting $\left[J^{2}\right]\approx g^{2}/\sqrt{K}$ and ${\left[J\right]}^{2}=J_{0}^{2}/K$ gives

$$\begin{array}{c} {}[{\left\langle \eta \right\rangle }^{2}]-{\left[\left\langle \eta \right\rangle \right]}^{2}\approx g^{2}\sqrt{K}[{\left\langle \phi \right\rangle }^{2}]\;. \end{array}$$ (17)

Define

$$\begin{array}{c} m=[\langle \phi \rangle ]\;,\;\;q=[{\left\langle \phi \right\rangle }^{2}]\;, \end{array}$$ (18)

which are the mean-field approximations for the mean and quenched fluctuations of the response *ϕ*. Additionally, define the autocorrelation function of *x* as

$$\begin{array}{c} \sigma \left(\tau \right)=[\langle x(t)x(t+\tau )\rangle ]-u^{2}\;, \end{array}$$ (19)

where *u* is the mean-field approximation for the mean of the total current, $\bar{x}$, namely

$$\begin{array}{c} u=I_{0}-J_{0}\sqrt{K}m\;. \end{array}$$ (20)

A similar calculation to Eq (16) shows that the autocorrelation of the synaptic input *R*_*η*_(*τ*) is related to that of the responses through

$$\begin{array}{c} \begin{array}{cc} R_{\eta }\left(\tau \right) & =[\langle (\eta (t)-\langle \eta \rangle )(\eta (t+\tau )-\langle \eta \rangle )]\\ & =g^{2}\sqrt{K}([\langle \phi (x(t))\phi (x(t+\tau ))\rangle ]-q)\;, \end{array} \end{array}$$ (21)

which is used when calculating the temporal correlations in the model. Our goal is to compute *m*, *q*, and *σ*(*τ*) self-consistently. Other quantities of interest, such as *R*_*η*_(*τ*), the decorrelation rate *β* and the fraction of active neurons *f* will fall out of the solution naturally, as we shown below.

The total current can be decomposed into

$$\begin{array}{c} x\left(t\right)=u+gK^{1/4}\sqrt{q}\;z+\sqrt{\sigma_{0}-g^{2}\sqrt{K}q}\;\xi \left(t\right)\;. \end{array}$$ (22)

The first term represents the mean total input, *z* is a static random variable that embodies the influence of quenched disorder, and *ξ* is a time-dependent variable with no quenched variance. We have used *σ*_0_ as a shorthand for *σ*(0), which is the total variance of *x*. With $g^{2}\sqrt{K}q$ as the quenched variance of *x* (or equivalently that of *η*), the prefactor $\sigma_{0}-g^{2}\sqrt{K}q$ is the remaining temporal variance of *x*.

There are two crucial differences between the expression above and that of the standard balanced models. First, unlike the mean-field decomposition in the standard balanced state, the input current *I*_0_, which enters *u*, is order 1. Second, the quenched variance is $g^{2}\sqrt{K}q$ as opposed to *g*^2^*q*. This follows form the fact that the variance of the connectivity is $\sqrt{K}$ larger in our model than in the conventional balanced state. These two differences result in distinct scaling properties of the mean-field variables (Fig 5).

From Eqs (18) and (20), we obtain

$$\begin{array}{c} u=-J_{0}\sqrt{K}\int_{-\infty }^{\infty }\;Dz^{\prime }\;\phi (u+\sqrt{\sigma_{0}}\;z^{\prime })+I_{0}\;, \end{array}$$ (23)

and

$$\begin{array}{c} q=[{\left\langle \phi \right\rangle }^{2}]=\int_{-\infty }^{\infty }Dz\;{\left(\int_{-\infty }^{\infty }D\xi \;\phi (u+gK^{1/4}\sqrt{q}\;z+\sqrt{\sigma_{0}-g^{2}\sqrt{K}q}\;\xi )\right)}^{2}\;. \end{array}$$ (24)

where integrals over temporal and quenched components of *x* are expressed as Gaussian integrals with $Dz=e^{-z^{2}/2}/\sqrt{2\pi }$ and likewise for *Dz*′ and *Dξ*. Given *σ*_0_, which remains to be found, the two equations above can be solved for *u* and *q* self-consistently.

In calculating *σ*_0_ and the full autocorrelation function of the dynamics, it is helpful to first define *δx* = *x* − *u* and form the correlated variables $\delta x\left(t\right)=\sqrt{\left|\sigma \right|}\;z+\sqrt{\sigma_{0}-\left|\sigma \right|}\;\xi$ and $\delta x\left(t+\tau \right)=\sqrt{\left|\sigma \right|}\;z+\sqrt{\sigma_{0}-\left|\sigma \right|}\;\xi^{\prime }$, assuming *σ* ≥ 0. Then, it is straightforward to show that

$$\begin{array}{c} \begin{array}{cc} \ddot{\sigma }\left(\tau \right) & =\sigma \left(\tau \right)-g^{2}\sqrt{K}[\langle \phi (x(t))\phi (x(t+\tau ))\rangle ]\\ & =\sigma -g^{2}\sqrt{K}\int_{-\infty }^{\infty }\;Dz\;{\left(\int_{-\infty }^{\infty }\;D\xi \;\phi (u+\sqrt{\left|\sigma \right|}\;z+\sqrt{\sigma_{0}-\left|\sigma \right|}\;\xi )\right)}^{2}\;, \end{array} \end{array}$$ (25)

with initial conditions *σ*(0) = *σ*_0_ and $\dot{\sigma }=d\sigma /d\tau =0$ at *τ* = 0. Together, Eqs (23), (24) and (25) form a closed system that can be used to determine *u*, *q* and *σ*(*τ*), and consequently *m* and *R*_*η*_(*τ*).

Solutions to Eqs (23)–(25) are computed numerically by evolving the dynamics of *σ* in time for an initial choice of *u* and *σ*_0_. Using the bisection algorithm, *σ*_0_ (and as a result *u*) is iteratively adjusted until the solution *σ* ceases to violate the conditions of the autocorrelation function, $\dot{\sigma }\leq 0$ and *σ* ≥ 0 for all *τ*. Alternatively, *σ*_0_, but not the full autocorrelation, could be determined through arguments that rely on conservation of energy in Eq (25) (see [16, 17, 21]). The converged values for *u* and *σ*_0_ can be used to calculate *m* (Fig 5A) and the fraction of active neurons (Fig 5B) using

$$\begin{array}{c} m=\frac{I_{0}-u}{J_{0}\sqrt{K}}\;,\;\;f=\frac{1}{2}(1+\text{erf}(\frac{u}{\sqrt{2\sigma_{0}}}))\;. \end{array}$$ (26)

The expression for *m* follows from Eq (20), while *f* is obtained by performing an average over the distribution of *x*, a Gaussian with mean *u* and total variance *σ*_0_. Additionally, with *q* obtained from Eq (24), we can insert *σ*(*τ*) into the right-hand-side of Eq (25) and solve for *R*_*η*_(*τ*) (Fig 5C) using Eq (21). Lastly, *β* (Fig 5D) is calculated from

$$\begin{array}{c} \beta =\frac{R_{\eta }\left(0\right)}{\int_{0}^{\infty }\;d\tau \;R_{\eta }\left(\tau \right)}\;. \end{array}$$ (27)

For a Heaviside step function, $\bar{\phi }=f$, and thus both $\bar{\phi }$ and *f* scale as $∼1/\sqrt{K}$. From the expression for *f* in Eq (26) and using the asymptotic form of the error function, it follows that, to leading order, $\left|u\right|/\sqrt{\sigma_{0}}∼\sqrt{\text{log}\;K}$.

### Numerical simulations

Numerical simulations of the network were performed using Euler integration with *τ*_*x*_ = 1, time-steps less than 0.05, and simulation time of *T* = 1000 or longer. Other network parameters are included in the figure captions. The code (written in Julia v1.3.0) is publicly available at https://github.com/raminkhajeh/sparse-balance.

The mean-field equations were implemented in Julia. Integrals over Gaussian random variables were carried out using adaptive quadrature. The dynamic equation for *σ*(*τ*) was evolved using the Tsitouras 5/4 Runge-Kutta method. The integral underneath the autocorrelation function, used in the definition of *β*, was carried out using trapezoidal integration.

## Supporting information

S1 Fig Asynchronous irregular activity in the sparse balance model with binary weights. (PDF)

S2 Fig Comparison of low- and high-variance networks with Gaussian connectivity. (PDF)

S3 Fig Equivalence between dilute and full connectivity. (PDF)

S4 FigSlow decay in *ψ*, defined as $\bar{\phi^{2}}/\bar{\phi }$.(PDF)

S5 Fig Sparse balance responds nonlinearly to input current. (PDF)

S6 Fig Recurrent synaptic input is described by a gamma distribution. (PDF)

S7 Fig Scaling of temporal and quenched variances. (PDF)
