# Supplementary material for: Sparse balance: Excitatory-inhibitory networks with small bias currents and broadly distributed synaptic weights
Source: PLoS Comput Biol. 2022 Feb 9;18(2):e1008836. doi: 10.1371/journal.pcbi.1008836 (PMC8827417; doi:10.1371/journal.pcbi.1008836)
Supplement: S1 Fig — (PDF) [file pcbi.1008836.s001.pdf]

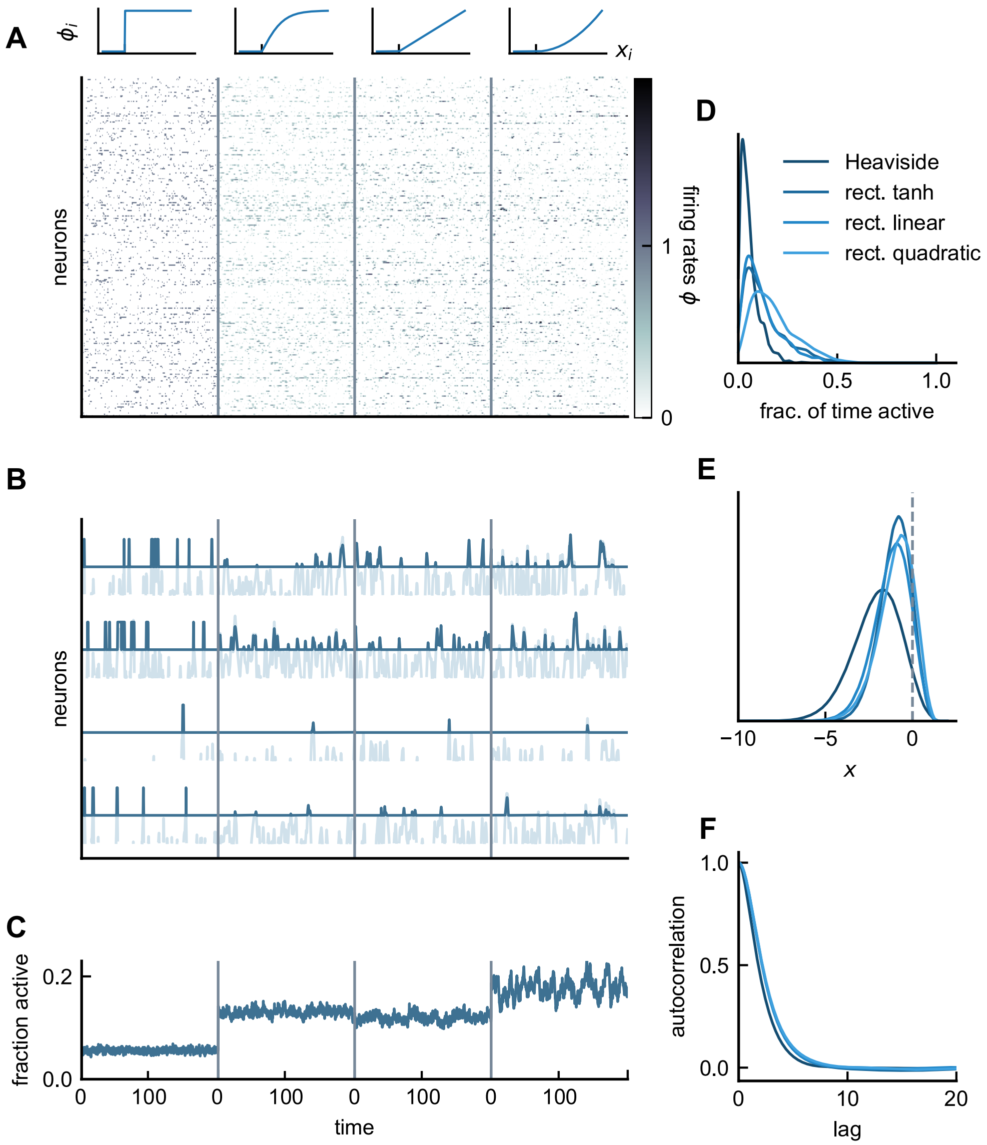

S1 Fig. Asynchronous irregular activity in the sparse balance model with binary weights. Same as Fig 3, except with a Bernoulli connectivity distribution of mean $J_0/\sqrt{K}$. The variance of a Bernoulli distribution is then $J_0\left(1 - J_0/\sqrt{K}\right)/\sqrt{K} = g^2/\sqrt{K}$ so, as in the other high-variance models, the mean and variance are both of order $1/\sqrt{K}$. (Model parameters: $J_0 = 2$, $g = \sqrt{J_0\left(1 - J_0/\sqrt{K}\right)}$, $I_0 = 1.5$, $N = K = 1000$).
