# Supplementary material for: Sparse balance: Excitatory-inhibitory networks with small bias currents and broadly distributed synaptic weights
Source: PLoS Comput Biol. 2022 Feb 9;18(2):e1008836. doi: 10.1371/journal.pcbi.1008836 (PMC8827417; doi:10.1371/journal.pcbi.1008836)
Supplement: S2 Fig — (PDF) [file pcbi.1008836.s002.pdf]

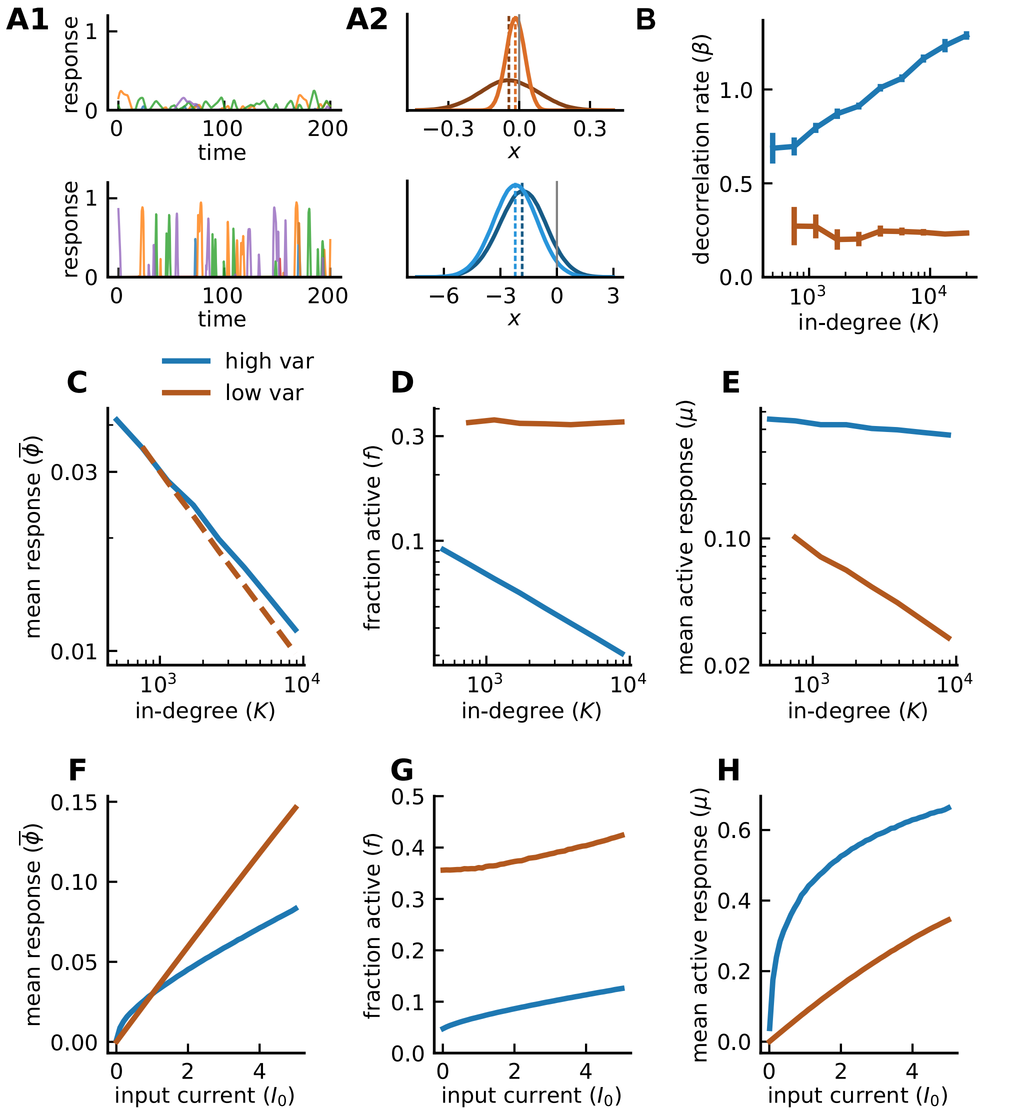

S2 Fig. Comparison of low- and high-variance networks with Gaussian connectivity. A1, C-H) Same as Fig 1 but with Gaussian-distributed synaptic weights, likewise for **A2** and Fig 2 and for **B** and Fig 4B. (Model parameters: $J_0 = 3$ for high variance and 1.1 for low variance, $g = 2$, $J_{ij} \sim$ Gaussian, $N = K$, $\phi = [\tanh]_+$)
