# Supplementary material for: Sparse balance: Excitatory-inhibitory networks with small bias currents and broadly distributed synaptic weights
Source: PLoS Comput Biol. 2022 Feb 9;18(2):e1008836. doi: 10.1371/journal.pcbi.1008836 (PMC8827417; doi:10.1371/journal.pcbi.1008836)
Supplement: S3 Fig — (PDF) [file pcbi.1008836.s003.pdf]

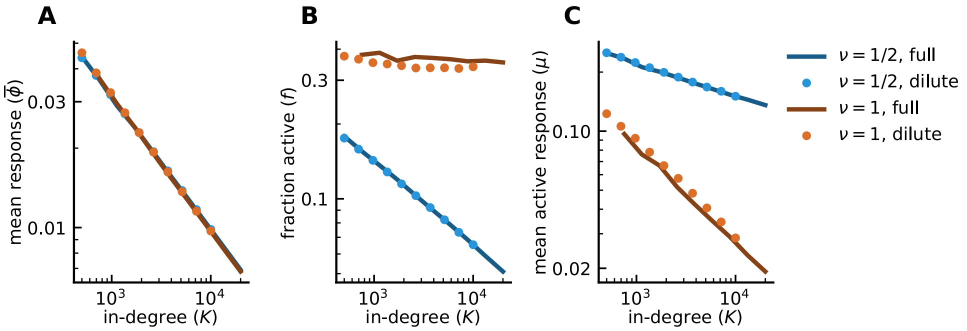

S3 Fig. Equivalence between dilute and full connectivity. A-C) Same as Fig 1C-E, but with the addition of results from a dilutely-connected network (dots). With dilute connectivity, the source of variability in the connections are twofold: each neuron receives inputs from, on average, K other randomly chosen neurons out of the total N network neurons; additionally, each existing connection is drawn from a distribution of mean $J_0/\sqrt{K}$ and variance $g^2/\sqrt{K}$. In the fully-connected case, each neuron receives input from all other neurons with connections drawn from a distribution of mean $J_0/\sqrt{N}$ and variance $g^2/\sqrt{N}$. Note that in the main text, we considered fully-connected networks and denoted the mean and variance by $J_0/\sqrt{K}$ and $g^2/\sqrt{K}$ with $K = N$. (Model parameters: $g = J_0 = 2$, $I_0 = 1$, $J_{ij} \sim$ gamma, $\phi = [\tanh]_+$; $N = K$ in full (solid), $N = 20000$ in dilute (dotted)).
