# Supplementary material for: Sparse balance: Excitatory-inhibitory networks with small bias currents and broadly distributed synaptic weights
Source: PLoS Comput Biol. 2022 Feb 9;18(2):e1008836. doi: 10.1371/journal.pcbi.1008836 (PMC8827417; doi:10.1371/journal.pcbi.1008836)
Supplement: S4 Fig — (PDF) [file pcbi.1008836.s004.pdf]

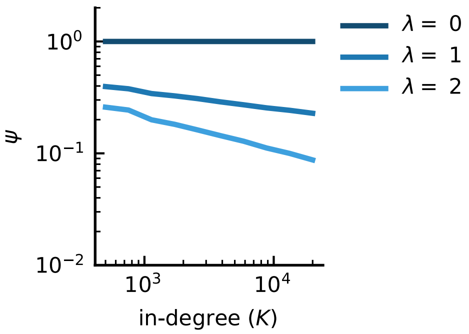

S4 Fig. Slow decay in ψ, defined as $\overline{\phi^2}/\overline{\phi}$. The scaling of ψ informs us about the effect on our analysis of using λ values other than zero. Generally, the variance of the synaptic input is $\text{var}(\eta) = K\text{var}(J)\overline{\phi^2} \sim K^{1-\nu}\psi\overline{\phi}$, where the mean response scales as $\overline{\phi} \sim 1/\sqrt{K}$. Assuming a Heaviside response function, where ψ is strictly one, allowed us to conclude that $\nu = 1/2$ is the only solution with finite fluctuations as K grows. This result assumes that ψ is independent of K. For $\lambda > 0$, we find numerically that, while not exactly one, ψ exhibits a slow decay in K. (Model parameters: $g = J_0 = 2$, $I_0 = 1$, $J_{ij} \sim$ gamma, $N = K$, $\phi = [x]_+^\lambda$)
