# Supplementary material for: Sparse balance: Excitatory-inhibitory networks with small bias currents and broadly distributed synaptic weights
Source: PLoS Comput Biol. 2022 Feb 9;18(2):e1008836. doi: 10.1371/journal.pcbi.1008836 (PMC8827417; doi:10.1371/journal.pcbi.1008836)
Supplement: S5 Fig — (PDF) [file pcbi.1008836.s005.pdf]

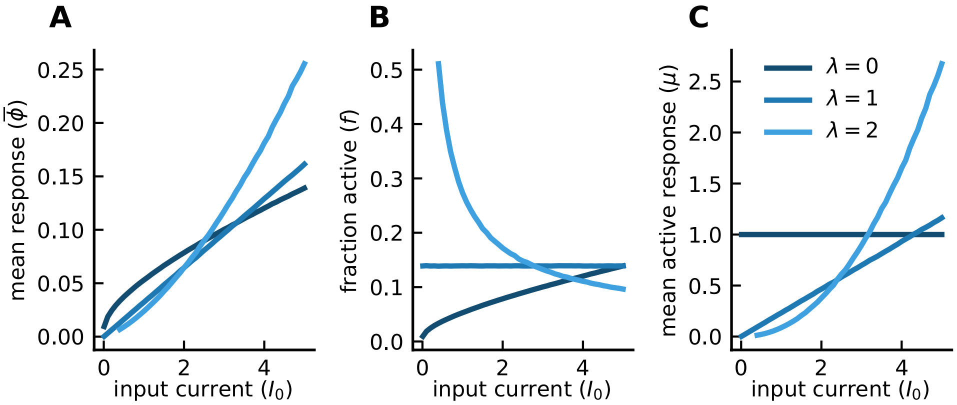

S5 Fig. Sparse balance responds nonlinearly to input current. A) Mean response, $\overline{\phi} = f\mu$, increases nonlinearly with input current. This relationship depends on the shape of the neuronal response function: $\lambda < 1$ and $\lambda > 1$ give rise to sublinear and supralinear mean responses, respectively. **B-C)** Fraction, f, and mean response, μ, of the active neurons versus input current. With rectified linear ($\lambda = 1$), this fraction remains constant since x can be rescaled by I_0^{-1} without changing the shape of the x distribution; this feature also makes the mean response linear. The nonlinear trend in sparsity switches at $\lambda = 1$. The fraction active f increases with I_0 at an ever-decreasing rate with $\lambda > 1$, while the opposite is true for $\lambda < 1$. For $\lambda > 1$, with stronger feedforward excitation, threshold crossings that result in sufficiently large responses become amplified. This amplification produces a large, supralinear μ, which in turn comes at the cost of sparsening the population activity with I_0. For the Heaviside ($\lambda = 0$), responses are binary, so $\mu = 1$ independent of I_0 and $\overline{\phi} = f$. (Model parameters: $g = J_0 = 2$, $J_{ij} \sim$ gamma, $\phi = [x]_+^\lambda$, $N = K = 1000$)
