# Supplementary material for: Sparse balance: Excitatory-inhibitory networks with small bias currents and broadly distributed synaptic weights
Source: PLoS Comput Biol. 2022 Feb 9;18(2):e1008836. doi: 10.1371/journal.pcbi.1008836 (PMC8827417; doi:10.1371/journal.pcbi.1008836)
Supplement: S6 Fig — (PDF) [file pcbi.1008836.s006.pdf]

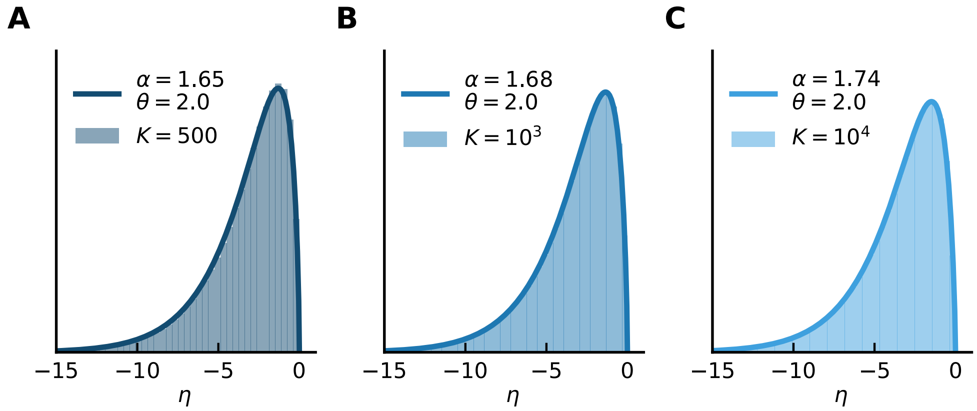

S6 Fig. Recurrent synaptic input is described by a gamma distribution. A-C) The distribution of the synaptic input η (across population and time) with a Heaviside response nonlinearity for three different values of K (shaded histograms). The solid line is the gamma distribution in Eq (10) with scale parameter $\theta = g^2/J_0$ and shape parameter $\alpha = f\sqrt{K}J_0^2/g^2$, where f is the measured sparsity in the simulations, and J_0, g, K are network parameters. The distribution accurately matches the histograms. Note that, due to the high J-variance in the sparse balance model, the distributions of synaptic input hardly change as K increases. (Model parameters: $g = J_0 = 2$, $I_0 = 1$, $J_{ij} \sim$ gamma, $\phi =$ Heaviside, $N = K$)
