# Supplementary material for: Sparse balance: Excitatory-inhibitory networks with small bias currents and broadly distributed synaptic weights
Source: PLoS Comput Biol. 2022 Feb 9;18(2):e1008836. doi: 10.1371/journal.pcbi.1008836 (PMC8827417; doi:10.1371/journal.pcbi.1008836)
Supplement: S7 Fig — (PDF) [file pcbi.1008836.s007.pdf]

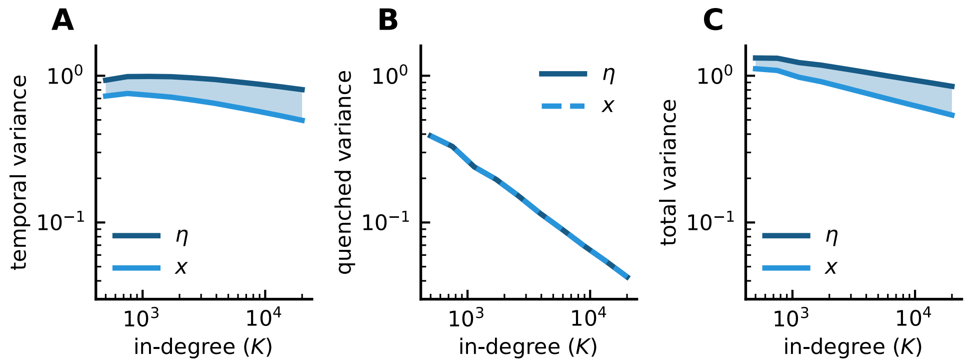


**S7 Fig. Scaling of quenched and temporal variances. A)** Temporal variances of the synaptic input $\eta$ and the total current $x$ remain roughly order 1. Shaded area highlights the difference between the two curves. **B)** Quenched variance of $\eta$ and $x$ are the same and decay as $\sim 1/\sqrt{K}$ (fit to data yields $1/K^{0.61}$). **C)** Total variance is the sum of temporal and quenched variances. Note the slightly faster decay in the total variance of $x$ as opposed to the total variance of $\eta$ (shaded area grows). Due to low-pass filtering, $x$ cannot keep up with the faster fluctuations in $\eta$, reducing its variance. The ratio between these two curves is plotted in Fig 4C. (Model parameters: $g = J_0 = 2$, $I_0 = 1$, $J_{ij} \sim$ gamma, $N = K$, $\phi = [\tanh]_+$.)
